# Condom use at last sexual relationship among adolescents of Santiago Island, Cape Verde, - West Africa

**DOI:** 10.1186/1742-4755-9-29

**Published:** 2012-11-15

**Authors:** Carlos Mendes Tavares, Néia Schor, Vitor E Valenti, Paula Yuri Sugishita Kanikadan, Luiz Carlos de Abreu

**Affiliations:** 1 Universidade da Integração Internacional da Lusofonia Afro-Brasileira, Setor de Estudo: Gestão da Informação no Setor Público e Métodos Quantitativos, Av. Universidade, 2995, Fortaleza, CE, 60020-181, Brasil; 2 Department of Mother and Child Health, School of Public Health – University of São Paulo, Av. Dr. Arnaldo, 715, São Paulo, SP 01246-904, Brazil; 3 Department of Speak and Hearing Pathology. Faculty of Phylosofy and Sciences, Paulista State University, UNESP, A. Hygino Muzzi Filho, 737, Marília, SP 17525-900, Brazil; 4 Researcher collaborator at Emílio Ribas Public Health Museum, Av. Dr. Arnaldo, 715, São Paulo, SP 01246-904, Brazil

**Keywords:** Adolescent health, Condom, Condom use, Sexual and reproductive health, Cape Verde, West Africa

## Abstract

**Objective:**

To estimate factors associated with condom use at last sexual intercourse among adolescents.

**Methods:**

Cross-sectional study of a representative sample of 368 sexually active adolescents aged 13–17 years from eight public high schools on Santiago Island, Cape Verde, 2007. The level of significance was 5.0% obtained from logistic regression, considering the association between condom use and socio-demographic, sexual and reproductive variables.

**Results:**

The prevalence of condom use at last sexual intercourse was 94.9%. Factors associated with condom use at last sexual relationship were: non-Catholic religion (OR=0.68, 95%CI: 0.52; 0.88) and affective-sexual partnership before the interview (OR=5.15, 95%CI: 1.79; 14.80).

**Conclusions:**

There was a high prevalence of condom use at last sexual intercourse of adolescents.

## Introduction

A Cape Verdean study [1] points to a relative increase in fecundity below 20 years of age and, inversely, a reduction in all other age-groups. The same tendency has been occurring in other countries, both developed and under developing [2,3]. Social aspects, such as abandonment of school, the limits of professional opportunities and future planning are also emphasized as possible consequences of the increase in fecundity in this population [4]. The AIDS epidemic and adolescent pregnancy have brought to juvenile sexuality greater visibility. Condom use has been promoted to young people [5].

There is an expectation that condom use should be more frequent among young population rather than adults. This fact occurs due to the easy information and educative campaigns currently which permits them to have more access to condom than adults. This fact happens even when teenagers do not use them in all their sexual relationships. Some studies on the use of methods of contraceptive protection have shown that greater knowledge does not necessarily mean the adequate or consistent use from adolescents. Although there has been a considerable increase in the use of contraceptive methods among this population in the last few years, this is not enough to achieve the targets of sexual health policies for younger population [5,6].

Education on sexuality in the adolescence will influence on forthcoming events in life. This behavior means that this formation will reflect on the development of health, social and economic aspects. The use of condom prevents sexual transmission infections which might occur due to the many kinds of relationship from adolescents. Several studies have shown the degree of protection offered by the use of condom for each of the sexually transmitted diseases (STDs) [7,8]. The basis for the delineation of sexual and reproductive health policies for young population is the knowledge of the factors associated with the use of condom among adolescents [9].

The purpose of the present study was the estimation of the factors associated with condom use by adolescents at their last sexual relationship.

## Methods

This study was undertaken on Santiago Island, in Cape Verde, an archipelago composed of ten islands (nine of them inhabited), situated in the Atlantic Ocean, approximately 500 km West of the African Continent. Santiago is the largest Island in the archipelago and has the most numerous population (54%) of the country, about 234,940 inhabitants [10].

This research is part of a wider cross-sectional study. We considered factors associated with the beginning of sexual life of Santiago Island adolescents per sex. The research, undertaken into a representative sample of adolescents (from 13 to 17 years old) resident on the island, was carried out from January to March, 2007. Written informed consent was obtained from the patient for publication of this report and any accompanying images.

A probabilistic sampling technique was used in this study. The sample was divided in two phases: the stratification was chosen by aleatory drawing of schools per county in phase 1 (sampling per strata); the sampling per conglomerate represented phase 2. This sample was shared per school with randomized draw of grades (considering all schools randomly selected) per school year and subsequent roll of all students with characteristics of interest in each selected class. Altogether we have assessed eight public schools among those sixteen already existing in the counties of the island.

The sample size was calculated considering the percentage of sexually-active adolescents (83.6%) with a presumed sampling error of 3.0% and a 95.0% confidence level. The estimated sample size was 576 adolescents, to which was added 28.0% to compensate occasional loss, considering a school population of 25,618 pupils. The final sample resulted in 768 adolescents who attended secondary level at the public school from the 7th to the 12nd school year. There was no registration of pupils in this age group enrolled at private institutions. For this study, only the sexually active pupils (368) were taken into consideration. We excluded 400 adolescents that were not sexually active. There was no refusal or loss among the 368 active pupils. We considered only 368 subjects for the data presentation, since they were sexually active.

The inclusion criteria in this study were: age between 13 and 17 years old; students must be regularly registered at a public secondary school; and adolescents must have already commenced sexual life.

A self-applied questionnaire (Additional file 1) was employed in this research, with 75 closed questions requiring social-demographic and behavior data. All adolescents replied to the questionnaire in the classroom. The average time to answer the questionnaire was 50 minutes. The dependent variable in the study was the use of male condom. Adolescents were asked, “Did you use a condom in your last sexual activity?”

The independent variables considered were: social-demographic – age, sex, schooling, marital status, religion and municipality of origin; affective-sexual variables: has already dated, age and partner at first sexual relationship; and behavioral variables – smoking habit, consumption of alcohol and assess to the media (radio, TV).

Anderson-Darling normality test was used to assess the employed variables. The descriptive analysis was presented by means of proportions, means and standard deviation. The odds ratio (OR) was calculated by means of non-conditional logistic regression. Significance was set at 5%.

The data were analyzed hierarchically: at the first level, social-demographic variables, and the behavioral, at the second level.

All the variables with a p value ≤ 0.20 at bivariate logistic regression were included in the complete model. In the final model, there was a maintenance of all the variables whose association with the use of condom persisted at a level ≤ 0.05.

The research project was undertaken in accordance with the norms of Resolutions no.196/1996 and no. 251/1997 of the National Health Council from Brazilian Ministry of Health. This study was approved by the Committee on Ethics in Research of the Public Health School, University of São Paulo. Finally, the research was financed by the post-graduate student-scholarship program, PEC-PG/Capes.

## Results

The average age of male adolescents at the beginning of their sexual life was 14 (SD=2.0) years old, and of female adolescents, 15 (SD=1.6). Approximately 65.6% (252) of male adolescents and 30.2% (116) of females declared that they had already begun their sexual life, with a statistically significant difference (p<0.001).

Considering the 368 adolescents who stated they had begun their sexual life, 69.3% of them reported that they used some kind of contraceptive method at their last sexual relationship. The most utilized contraceptive methods were the exclusive use of condom (94.9%) and pill usage (26.4%).

Younger male adolescents (those aged between 13 and 14 years old) reported more condom use than female adolescents.

Catholic adolescents presented more frequent use of condom at their last sexual relationship (96%, for both sex) than non-Catholics.

The factors associated with the use of condom, with their respective ORs and 95% CI, as well as their levels of significance (p), are presented in Tables 1, 2 and 3.

**Table 1:** Estimates of multiple logistic regression model for condom use in the last recent sexual relationship, according to selected variables

**Variable**	**Use of condom**		
	**OR**	**Variable**	**OR**
**Sex**		**Sex**	
Female (reference)		Female (reference)	
Male	0.85	Male	0.85
**Condom use** at first sexual		**Condom use** at first sexual	
Yes	2.96	Yes	2.96
No (reference)		No (reference)	
**Already have STD**		**Already have STD**	
Yes	0.09	Yes	0.09
No (reference)		No (reference)	
**Schooling**		**Schooling**	
≤ 10 years (reference)		≤ 10 years (reference)	
> 10 years	0.55	> 10 years	0.55
**Age group**		**Age group**	
≤ 14 years (reference)		≤ 14 years (reference)	
> 14 years	3.33	> 14 years	3.33
**Already dating**		**Already dating**	
Yes	5.05	Yes	5.05
No (reference)		No (reference)	
**Municipality of origin**		**Municipality of origin**	
Others (reference)		Others (reference)	
Beach	1.60	Beach	1.60
**Religion**		**Religion**	
Catholic (reference)		Catholic (reference)	
Others (evangelical and spiritualist)	0.68	Others (evangelical and spiritualist)	0.68
**Marital status**		**Marital status**	
Single (reference)		Single (reference)	
Cohabiting with partner	0.18	Cohabiting with partner	0.18
Dating	0.68	Dating	0.68
**Has television**		**Has television**	
No (reference)		No (reference)	
Yes	0.86	Yes	0.86
**Has radio**		**Has radio**	
No (reference)		No (reference)	
Yes	1.81	Yes	1.81
**Consume of alcoholic beverages**		**Consume of alcoholic beverages**	
No (reference)		No (reference)	
Yes	1.53	Yes	1.53
**Partner at first sexual experience**		**Partner at first sexual experience**	
Date (reference)		Date (reference)	
Friend	0.86	Friend	0.86
Relative	0.81	Relative	0.81
Husband or wife	0.24	Husband or wife	0.24
**Age at first sexual experience**		**Age at first sexual experience**	
11-12 years (reference)		11-12 years (reference)	
13 years or more	0.42	13 years or more	0.42

Santiago Island, Cape Verde, 2007. *p < 0.05 and **p < 0.20.

**Table 2:** Estimates of multiple logistic regression model for condom use in the last sexual relationship, for the complete model

**Variable**	**Use of condom**		
	**OR**	**p**	**CI 95%**
**Age group**			
≤ 14 years (reference)			
> 14 years	1.97	0.289	[0.56; 6.93]
**Dating?**			
Yes	5.04	0.011	[1.46; 17.45]
No (reference)			
**Religion**			
Catholic (reference)			
Others (evangelical and spiritualist )	0.67	0.005	[0.51; 0.89]
**Marital status**			
Single and dating (reference)			
Cohabiting with partner	0.17	0.084	[0.02; 0.27]
**Age at first sexual experience**			
11-12 years (reference)			
13 years or more	1.26	0.719	[0.36; 4.138]

Santiago Island, Cape Verde, 2007.

*Odds ratio:* adjusted by means of regression, including all variables of Table 1 with p < 0.20.

**Table 3:** Estimates of multiple logistic regression model of condom use at the last sexual relationship, for the final model

**Variable**	**Use of condom**		
	**OR**	**p**	**CI 95%**
**Dating?**			
Yes	5.15	0.002	[1.79; 14.80]
No (reference)			
**Religion**			
Catholic (reference)			
Others (evangelical and spiritualist )	0.68	0.004	[0.52; 0.88]

Santiago Island, Cape Verde, 2007.

*Odds ratio:* adjusted by means of regression, including all variables of Table 2 with p < 0.05.

The variables which had significant effect within the complete model were: has already dated and religion (Table 2). Those ones with p>0.05 were excluded from the model.

After the final multivariate analysis (Table 3), non-Catholic religion was a factor associated with the non-use of condom among adolescents at their last sexual relationship; on the other hand, the factor positively associated with use of condom was “having dated before”.

## Discussion

This research is a probabilistic and representative study, whose data have been analyzed by multivariate models, considering condom use and associated factors among adolescents. The population studied in this study was equivalent to 74% of all adolescents enrolled at secondary education in Cape Verde [11]. As a main finding, there was a great frequency of condom use in the last sexual relationship, which may be a result of programs prevention. We believe that differential by religion and affective-sexual partner in condom use is due to the need of protective actions to non-Catholic religious.

In this research some new variables were measured, which we will discuss below. However, some important variables which might explain the conclusion of the study [9,12] were not measured in this study. Another limitation of this research is the possible bias in the declaration related to gender expectations, that is, boys might have reported more experiences than girls [13]. Also, we did not assess unwanted pregnancy cases.

The high prevalence of the use of condom at last sexual relationship obtained at the present study is similar to that presented by the research data on demography and reproductive health at the population of Cape Verde, especially in female adolescents between 15 and 19 years old [12,14]. However, the prevalence found in the present research (94.9%) differs greatly in magnitude from those one obtained among juvenile population of Cape Verde (from 15 to 19 years old) [55.3%], and from the population of this age group in Santiago Island (48.4%) [14].

The low prevalence of estimated HIV in Cape Verde [15] (varying from 0.8% to 1.5% of the population) may be explained by the high frequency of the use of condom. However, although the prevalence of the epidemic in Cape Verde may be lower than in Sub-Saharan Africa countries, where the epidemic seems to have sustained (prevalence of 7.4% [5]), it is still a matter of concern for Cape Verdian public health.

Factors that may explain the frequency of the use of condom at last sexual relationship among adolescents of the island are: free distribution of condoms; the policy of cooperation with Brazil and Portugal regarding prevention; the treatment of sexually transmitted diseases (STDs) such as HIV/AIDS; and the implantation of the program of sexual and reproductive education in secondary schools.

According to similar national [16] and international [5,17] studies it is revealed that the negotiation of the use of condom during the sexual act encounter obstacles based on social patterns: men usually present more power of decision than women. These obstacles, thus, are usually faced by women, more vulnerable and emotionally evolved than men. This sensitive characteristic of women shall contribute to the non-use of condom and consequently, their unprotected sex, facilitating the infection of STDs/AIDS.

The understanding of the differences between men and women regarding the use of the condom needs cautious analysis. The choices exercised by men and women in their sexual lives are associated with the existence of gender differences related to conceptions about affective-sexual connection [18]. These conceptions are related to the need of protection [18], and are based on the difficulty of women in negotiating the use of condom. The status of the relationship also influences the choice of having sex with or without condom.

Our study corroborates the data of the studies discussed above, despite the evidences do not point to the vulnerability of women to the non-use of condom. However, the findings reinforce the association with the perception of greater risk for HIV or the previous occurrence of SDTs among female adolescents whose use of condom was less frequent.

Corroborating other studies in the literature, [12] we observed that religion and affective-sexual partner are important factors to be taken into consideration both in research and in the organization of prevention services and assistance. The distribution by religion coincides with that described in research projects on demography and reproductive health [13,18]. In these studies, Roman Catholics use condom more frequently, and the frequency was stronger in girls than in boys, despite the absence of statistical significance.

Religious affiliation influenced the use of condom in sexual relations: Catholic adolescents reported more frequent use than did those of other religions. These findings are important for the planning of strategies related to the use of contraceptive methods. These strategies might avoid undesired pregnancies and STDs, including AIDS.

On the other hand, another Cape Verdean study with focus on qualitative characteristics of adolescent population did not report [18] any association between condom use and religious affiliation. Nonetheless, religious category did not distinguished creeds.

In the present study the use of the condom at last sexual relation was much more frequent among adolescents who had an affective-sexual partner in the period prior to the research. These evidences differ from those observed in studies from other countries [16,19,20]. The international literature pointed that condom was partially replaced by other methods, such as oral or injection hormonal contraceptive methods. This might be explained by the fact that they had replaced condom as their contraceptive method of choice, despite adolescents interviewed have begun their sexual life outside a stable union and at an earlier age. Adolescents substituted condom use precisely because they were involved in a more stable and affective relationship at that moment. This form of relationship confirms a tendency of non use of condom when the relationship becomes more stable. It might be a proof of love, confidence and fidelity to the partner. It also might be a consequence of a protection feeling and monogamous fidelity.

The data of the present study suggest that male adolescents begin their sexual life before female, and that condom increases safety on the protection against STDs/HIV as well as undesirable pregnancy [21,22]. The asymmetries of sexual life beginning between men and women are based on differences of attitude, family and social expectancies in comparison to female and male conduct face to sexuality. Boys seem to be more stimulated to sexual initiation before marriage, while girls consider that this practice is not socially accepted [23].

Despite this study reinforces the idea of sexual life beginning at an earlier age, these evidences are opposite to what really happens in current life of adolescents from Cape Verde. In general these adolescents do not have adequate information [23]. More than 90% knew some kind of contraceptive method from the total population studied here – a significant level of information about the more familiar forms of contraception -, and the majority knew that they should use condom.

In conclusion, we show a high prevalence of condom use in the last sexual relationship. This fact might be a consequence of official programs in STDs/AIDS prevention. On the other hand, results regarding condom use according to religion and affective-sexual partner reveal the necessity of preventive actions directed to non-Catholic religious segments and to those who do not have an affective-sexual partner.

## Competing interests

The authors declare no conflict of interest.

## Authors’ contributions

CMY, NS, VEV, PYSK and LCA participated in the acquisition of data and revision of the manuscript. All authors conceived of the study and determined the design, CMT performed the statistical analysis. All authors interpreted the data and drafted the manuscript. All authors read and gave final approval for the version submitted for publication.

## Supplementary Material

Additional file 1 Questionnaire applied to the subjects. Click here for file
