# Supplementary material for: Condom use at last sexual relationship among adolescents of Santiago Island, Cape Verde, - West Africa
Source: Reprod Health. 2012 Nov 15;9:29. doi: 10.1186/1742-4755-9-29 (PMC3538512; doi:10.1186/1742-4755-9-29)
Supplement: Additional file 1 — Questionnaire applied to the subjects. [file 1742-4755-9-29-S1.doc]

| Questão | | Resposta | Código |
| --- | --- | --- | --- |
| 1 | Indique o seu sexo? | **1 ( ) Feminino** **2 ( ) Masculino** | **Q1** |
| 2 | Quantos anos você tem? | **1( ) 13 anos** **2( ) 14 anos** **3( ) 15 anos** **4( ) 16 anos****5( ) 17 anos** | **Q2** |
| 3 | Qual a sua nacionalidade? | 1( ) Caboverdiana  2( ) Estrangeira. Nome do país: ___________________ **3( ) Dupla** | **Q3** |
| 4 | Indique seu local de seu nascimento? | 1 ( ) Ilha. Nome da ilha: ___________________ **2 ( ) Estrangeiro. Nome do país:** ___________________ | **Q4** |
| 5 | Em que ano você está estudando atualmente? | 1( ) 7^o^ ano  2( ) 8^o^ ano  3( ) 9 ^o^ ano  4 ( ) 10^o^ ano  5( )11^o^ ano  6( ) 12^o^ ano | **Q5** |
| 6 | **Você já repetiu o ano escolar alguma vez?** | 0( ) Não  1( ) Sim, 1 vez  2( ) Sim, mais de 1 vez | **Q6** |
| 7 | **Qual período você estuda?** | 1( ) Manhã  2( ) Tarde | **Q7** |
| 8 | **Qual é o seu meio de subsistência?** | 1( ) Trabalho  2( ) A cargo da família que vive em Cabo Verde  3( ) A cargo da família que vive no estrangeiro  4( ) Outro. Qual? ___________________ | **Q8** |
| 9 | **Qual é o seu estado civil?** | 1( ) Solteiro(a)  2( ) Casado(a)  3( ) Vive com companheiro(a)  4( ) Namoro(a)  5( ) Separado (a)  6( ) Outro. Qual ? ___________________ | **Q9** |
| 10 | **Qual é a sua relação de parentesco com o chefe (responsável) pelo domicílio?** | 1( ) Chefe  2( ) Cônjuge  3( ) Filho (a)  4( ) Irmão/irmã  5( ) Neto(a)/bisneto(a)  6( ) Sobrinho (a)  7( ) Enteado (a)  8( ) Outra. Qual? ____________________  9( ) Sem parentesco | **Q10** |
| 11 | **A casa em que você mora é:** | 1( ) Própria  2( ) Arrendada  3( ) Cedida  4( ) Outra. Qual? ____________________ | **Q11** |
| 12 | **Quantas divisões tem a sua casa?** (sem contar com a cozinha e casa de banho): | ____________ divisão/divisões | **Q12** |
| 13 | **Destas divisões, quantas são para dormir ?** | **____________** | **Q13** |
| 14 | **Qual é a principal fonte de abastecimento da água que usam em seu domicílio?** | 1( ) Água canalizada da rede pública  2( ) Cisterna  3( ) Autotanque  4( ) Chafariz  5( ) Poço  6( ) Nascente  7( ) Outro. Especificar: _______________________ | **Q14** |
| 15 | **Tem cozinha?** | 0( ) Não  1( ) Sim | **Q15** |
| 16 | **A sua casa tem:** | 1( ) Casa de banho com retrete (sanita)  2( ) Casa de banho sem retrete (sanita)  3( ) Latrina (casa de banho fora de casa)  4( ) Sem casa de banho, sem retrete (sanita) e sem latrina | **Q16** |
| 17 | **Qual é a principal fonte de energia para preparação dos alimentos?** | 1( ) Carvão  2( ) Lenha  3( ) Petróleo  4( ) Gás  5( ) Eletricidade  6( ) Outra. Especificar: ___________________ | **Q17** |
| 18 | **Qual é a principal fonte de energia para iluminação?** | 1( ) Eletricidade  2( ) Petróleo  3( ) Gás  4( ) Outra. Especificar: ___________________ | **Q17** |
| 19 | **A sua casa tem rádio?** | 0( ) Não  1( ) Sim | **Q18** |
| 20 | **Tem televisão?** | 0( ) Não  1( ) Sim | **Q20** |
| 21 | **Tem frigorífico?** | 0( ) Não  1( ) Sim | **Q21** |
| 22 | **Tem automóvel?** | 0( ) Não  1( ) Sim | **Q22** |
| 23 | **Tem videocassete?** | 0( ) Não  1( ) Sim | **Q23** |
| 24 | **Qual o número total de pessoas que mora no seu domicílio (incluindo você e os seus pais, se morarem todos na mesma casa)?** | ___________________ pessoas | **Q24** |
| 25 | **NESTA QUESTÃO MARQUE COM X TODAS AS RESPOSTAS CORRETAS**.  **Com quem você mora?** | 1( ) Pai  2( ) Mãe  3( ) Madrasta  4( ) Padrasto  5( ) Irmão (s)  6( ) Irmã (s)  7( ) Avó  8( ) Avô  9( ) Tio (s)  10( ) Tia (s)  11( ) Companheiro (a)  12( ) Sogra  13( ) Sogro  14( ) Primo (s)  15( ) Sobrinho(s)  16( ) Amigo(a)  17( ) Filho (a)  18( ) Vizinho (a)  19( ) Outros. Especificar: ___________________ | **Q25** |
| 26 | **Qual é a idade do responsável (chefe) pelo domicílio?** | ________ anos | **Q26** |
| 27 | **Qual é o sexo do responsável (chefe) pelo domicílio?** | 1( ) Masculino  2( ) Feminino | **Q 27** |
| 28 | **Qual é o nível de ensino mais elevado que o responsável (chefe) pelo domicílio freqüentou ou freqüenta?** | 1( ) Alfabetização  2( ) Ensino básico integrado  3( ) Secundário  4( ) Curso médio  5( ) Curso superior | **Q 28** |
| 29 | **Qual é a sua religião?** | 1( ) Católica  2( )Evangélica  3( ) Espírita  4( ) Outra. Qual? ___________________  5( ) Não tenho religião, mas acredito em Deus  6( ) Não acredito em Deus | **Q 29** |
| 30 | **Você freqüenta sua religião ou algum culto?** | 0( ) Não  1( ) Sim | **Q 30** |
| 31 | **Você já namorou?** | 0( ) Não (**SE** **NÃO,** passa para a questão 37)  1( ) Sim, qual sua idade no primeiro namoro? ________ anos | **Q 31** |
| 32 | **Hoje você namora?** | 0( ) Não (**SE** **NÃO,** passa para a questão 37 – se for menina – ou para questão 39 – se for menino)  1( ) Sim, há quanto tempo? _____________________ | **Q 32** |
| 33 | **SE** **SIM,**  **Que idade tem o seu namorado(a)?** | ________ anos | **Q33** |
| 34 | **Ele/ela estuda?** | 0( ) Não (**SE** **NÃO,** passa para a questão 36)  1( ) Sim | **Q 34** |
| 35 | **SE** **SIM,**  **Que ano ele/ela estuda?** | 1( ) 7^o^ ano  2( ) 8 ^o^ ano  3( ) 9 ^o^ ano  4( ) 10 ^o^ ano  5( ) 11 ^o^ ano  6( ) 12 ^o^ ano ou mais | **Q 35** |
| 36 | **Indica qual é o principal meio de subsistência dele/dela?** | 1( ) Trabalho  2( ) A cargo da família residente em Cabo Verde  3( ) A cargo da família residente no estrangeiro  4( ) Outro, Qual?____________________ | **Q 36** |

SÓ PARA MENINAS (perguntas 37 e 38)

| 37 | **Você já teve a sua primeira menstruação?** | 0( ) Não (**SE** **NÃO,** passa para a questão 39)  1( ) Sim | **Q37** |
| --- | --- | --- | --- |
| 38 | **SE** **SIM,**  **Que idade você tinha quando teve a primeira menstruação?** | ______anos. | **Q38** |

PARA RAPAZES E MENINAS (perguntas de 39 a 78)

| 39 | **Você teve relação sexual alguma vez?** | 0( ) Não (**SE** **NÃO,** passa para a questão 56)  1( ) Sim | **Q39** |
| --- | --- | --- | --- |
| 40 | **Com quem você teve a sua primeira relação sexual?** | 1( ) Namorado(a)  2( ) Amigo(a)  3( ) Parente  4( ) Noivo(a)  5( ) Marido/esposa  6( ) Outro. Quem? ___________________ | **Q40** |
| 41 | **Foi de comum acordo entre os dois?** | 1( ) Sim  2( ) Não  3( ) Eu forcei a situação  4( ) Ele/ela forçou a situação  5( ) Outro motivo. Qual? ____________________________ | **Q41** |
| 42 | **Qual foi a emoção mais importante que você sentiu na sua primeira relação sexual?** | 1( ) Dor  2( ) Medo  3( ) Felicidade/amor  4( ) Prazer  5( ) Outra. Qual? ___________________  9( ) Não sei | **Q42** |
| 43 | **Você acha que a primeira relação sexual foi planejada ou simplesmente aconteceu?** | 1( ) Planejada  2( ) Aconteceu  3( ) Outro motivo. Qual? ______________  9( ) Não sei | **Q43** |
| 44 | **Quem foi a primeira pessoa para quem você contou sobre sua primeira relação sexual?** | 1( ) Minha mãe  2( ) Meu pai  3( ) Professor(a)  4( ) Meu/minha vizinho (a)  5( ) Amigo(a)  6( ) Outra pessoa. Quem? ___________________ | **Q44** |
| 45 | **Que idade você tinha quando teve sua primeira relação sexual?** | _________ anos (completo) | **Q45** |
| 46 | **Seus pais sabem (sabiam) que você tem (tinha) vida sexual?** | 0( ) Não  1( ) Sim  2( ) Só minha mãe sabia  3( ) Só meu pai sabia | **Q46** |
| 47 | **Quando você teve a primeira relação sexual, a maioria dos(as) seus(as) amigos(as) já tinham tido relações sexuais?** | 0( ) Não  1( ) Sim  9( ) Não sei | **Q47** |
| 48 | **Você conhecia métodos ou maneiras de evitar filhos ou de evitar doenças sexualmente transmissíveis (por exemplo: SIDA e outras) na sua primeira relação sexual?** | 0( ) Não  1( ) Sim  9( ) não sei/não me lembro | **Q48** |
| 49 | **NESTA QUESTÃO MARQUE COM X TODAS AS RESPOSTAS CORRETAS.**  **Quais os métodos ou maneiras de evitar filhos ou de evitar doenças sexualmente transmissíveis (por exemplo: SIDA e outras) que você já ouvia falar quando da sua primeira relação sexual?** | 1( ) Pílula  2( ) Camisinha  3( ) Injeção  4( ) Coito interrompido (gozar fora)  5( ) Tabelinha ou calendário  6( ) Outro. Qual? ___________________  7( ) Não ouviu falar  8( ) Abstinência (não ter relações sexuais)  9( ) DIU  99( ) Não sei/não me lembro | **Q49** |
| 50 | **Você usou algum método para evitar filhos ou doenças sexualmente transmissíveis (por exemplo: SIDA e outras) na primeira relação sexual?** | 0( ) Não (**SE** **NÃO,** passa para a questão 55)  1( ) Sim | **Q50** |
| 51 | **SE SIM,**  **Quem tomou a primeira iniciativa de usar este método?** | 1( ) Eu  2( ) Companheiro(a)  3( ) Nós dois  9( ) Não sei/não me lembro | **Q51** |
| 52 | **NESTA QUESTÃO MARQUE COM X TODAS AS RESPOSTAS CORRETAS.**  **Quais os métodos você utilizou na sua primeira relação sexual?** | 1( ) Pílula  2( ) Camisinha  3( ) Injeção  4( ) Coito interrompido (gozar fora)  5( ) Tabelinha ou calendário  6( ) Outro. Qual? ___________________  7( ) Não usou  8( ) Abstinência (não ter relações sexuais)  9( ) DIU  99( ) Não sei/não me lembro | **Q52** |
| 53 | **Você usou camisinha na sua primeira relação sexual?** | 0( ) Não (**SE** **NÃO,** passa para a questão 55)  1( ) Sim | **Q53** |
| 54 | **NESTA QUESTÃO MARQUE COM X TODAS AS RESPOSTAS CORRETAS**  **SE SIM,**  **Por que usou?** | 1( ) Para evitar a gravidez 2( ) Pode prevenir duplo propósito: gravidez e doenças sexualmente transmissíveis, incluindo SIDA3( ) Para evitar doenças sexualmente transmissíveis, incluindo SIDA 4( ) Não conhecia bem companheiro(a)  5( ) Outros motivos. Quais? ___________________  9( ) Não sei/não me lembro | **Q54** |
| 55 | NESTA QUESTÃO MARQUE COM X TODAS AS RESPOSTAS CORRETAS. **SE NÃO,**  **Por que não usou?** | 1( ) Não esperava ter relação sexual  2( ) Queria engravidar  3( ) Tinha vergonha  4( ) Conhecia bem o(a) companheiro(a)  5( ) Não gosta de usar **6( ) Outros motivos. Quais?** ___________________ 9( ) Não sei/não me lembro | **Q55** |
| 56 | **Você tem intenção de ter filho?** | 0( ) Não (**SE** **NÃO,** passa para a questão 58)  1( ) Sim | **Q56** |
| 57 | **NESTA QUESTÃO MARQUE COM X TODAS AS RESPOSTAS CORRETAS (SÓ PARA MENINAS).**  **SE SIM,**  **Quais os motivos que levam você a ter intenção de ter filho?** | 1( ) Forte desejo de ter o filho  2( ) Uma companhia para a velhice  3( ) Quero ter filhos  4( ) Quero ter mais filhos  5( ) Para agradar ao marido/companheiro  6( ) Outros motivos. Quais? ___________________  9( ) Não sei | **Q57** |
| 58 | **QUESTÃO MARQUE COM X TODAS AS RESPOSTAS CORRETAS.**  **SE NÃO,**  **Quais os motivos que levam você a não ter intenção de ter filho?** | 1( ) Medo do contágio da criança  2( ) Tem infecção HIV  3( ) Já tem filho/s  4( ) Problemas econômicos  5( ) Para agradar ao marido/companheiro  6( ) Outros motivos, Quais? ___________________  9( ) Não sei | **Q58** |
| 59 | **Você conhece alguém próximo que já realizou aborto?** | 0( ) Não (**SE** **NÃO,** passa para a questão 61)  1( ) Sim | **Q59** |
| 60 | **SE SIM,**  **Que idade tinha esta pessoa?** | ________ anos | **Q60** |
| 61 | **Você ouviu falar de alguma doença sexualmente transmissível?** | 0( ) Não  1( ) Sim. Quais? ______________________________________ | **Q61** |
| 62 | **Você conhece as formas para evitar a contaminação de doenças sexualmente transmissíveis ou SIDA?** | 0( ) Não (**SE** **NÃO,** passa para a questão 64)  1( ) Sim | **Q62** |
| 63 | **NESTA QUESTÃO MARQUE COM X TODAS AS RESPOSTAS CORRETAS.**  **SE SIM,**  **Quais são as formas de evitar as doenças sexualmente transmissíveis, incluindo SIDA?** | 1( ) Higiene pré-coito  2( ) Usar camisinha  3( ) Escolha de parceiros sexuais  4( ) Evitar a promiscuidade/ter um único parceiro  5( ) Higiene pós-coito  6( ) Outra forma. Qual? ___________________  7( ) Uso de seringas descartáveis  9( ) Não sei | **Q63** |
| 64 | **NESTA QUESTÃO MARQUE COM X TODAS AS RESPOSTAS CORRETAS.**  **SE NÃO,**  **Quais são as formas comprovadas de transmissão da SIDA?** | 1( ) Beijar a boca de uma pessoa infectada pelo vírus da SIDA  2( ) Ter relação vaginal, sem camisinha, com uma pessoa infetada pelo vírus da SIDA  3( ) Ser picado por mosquito que picou uma pessoa infectada pelo vírus da SIDA  4( ) Compartilhar seringas e agulhas com outras pessoas que estejam infectadas pelo vírus da SIDA  5( ) Receber transfusão com sangue contaminado pelo vírus da SIDA  6( ) Doar sangue  7( ) Durante a gravidez da mãe infectada pelo vírus da SIDA para o seu bebê, através da placenta  9( ) Não sei | **Q64** |
| 65 | **Se considerar a afirmação verdadeira, marque um V; se considerar a afirmação falsa, marque um F; se não sabe responder, marque um N. Para usar camisinha corretamente na relação sexual deve-se:** | 1  Colocá-la antes de penetração  2  Colocá-la apenas na hora de ejaculação (gozo)  3  Nunca deixar um espaço na ponta | **Q65** |
| 66 | **NESTA QUESTÃO MARQUE COM X TODAS AS RESPOSTAS CORRETAS**  **Que tipo de atendimento você já recebeu no posto ou centro de saúde?** | 1( ) Tratamento de doenças transmissíveis pelo sexo  2( ) Informações sobre como evitar a gravidez  3( ) Recebimento de método para evitar a gravidez  4( ) Informações sobre doenças sexualmente transmissíveis (dst) e sida  5( ) Consulta pré-natal ou pós-parto  6( ) Outro tipo de atendimento não relacionado com sexualidade, gravidez ou dst/sida  7( ) Nunca esteve no posto de saúde | **Q66** |
| 67 | **NESTA QUESTÃO MARQUE COM X TODAS AS RESPOSTAS CORRETAS**  **Qual o principal motivo pelo qual você NÃO IRIA A UM POSTO DE SAÚDE para problemas relacionados com sexualidade, DST/SIDA ou gravidez?** | 1( ) O tempo de espera no posto é muito longo  2( ) A equipe de saúde não trata os adolescentes com respeito ou atenção  3( ) Geralmente, o posto de saúde não tem os medicamentos necessários  4( ) Pode ser que eu seja atendido por alguma pessoa que me conheça ou a minha família  5( ) Estes problemas são mais bem discutidos fora do posto de saúde  6( ) Tenho vergonha  7( ) Outro motivo. Qual?_______________________________  9( ) Não sei | **Q67** |
| 68 | **NESTA QUESTÃO MARQUE COM X TODAS AS RESPOSTAS CORRETAS**  **Nos últimos anos, se você recebeu informações FORA DE CASA sobre sexualidade, drogas, prevenção das doenças transmitidas pelo sexo (DST) e da SIDA e como evitar a gravidez, como isso aconteceu?** | 1( ) Com meu pai  2( ) Com minha mãe  3( ) Com outros parentes  4( ) Com amigos  5( ) Com profissionais do posto de saúde  6( ) Televisão, radio, jornais ou revistas  7( ) Outra forma. Qual? ___________________  8( ) Não recebi informação desse tipo fora de casa  9( ) Não sei/não me lembro | **Q68** |
| 69 | **NESTA QUESTÃO MARQUE COM X TODAS AS RESPOSTAS CORRETAS**  **Nos últimos anos, se você recebeu informações NA ESCOLA sobre sexualidade, drogas, prevenção das doenças transmitidas pelo sexo (DST) e da SIDA e como evitar a gravidez, como isso aconteceu?** | 1( ) Durante as aulas, (em quais disciplinas?) ___________________  2( ) Individualmente com um professor  3( ) Conversando com os amigos da escola  4( ) Outra forma (qual?) ______________________________  5( ) Nunca tive informação deste tipo na escola  9( ) Não sei | **Q69** |
| 70 | **Você tem medo de pegar alguma doença transmitida pelo sexo (DST/SIDA)?** | 0( ) Não  1( ) Sim | **q70** |
| 71 | **Quanto tempo você poderá demorar para ir ao serviço de saúde mais próximo do seu domicílio?** | **Minutos**  0( ) 0 a 14  1( ) 15 a 29  2( ) 30 a 44  3( ) 45 e+ | **q71** |
| 72 | **Que meio de transporte você utiliza, geralmente, para ir ao serviço de saúde mais próximo?** | 0( ) A pé  1( ) Carro privado  2( ) Autocarro (coletivo)  3( ) Outro. Qual? ___________________ | **q72** |
| 73 | **Você fuma?** | 0( ) Não (**SE** **NÃO,** passa para a questão 75)  1( ) Sim. Com que idade? _______anos | **q73** |
| 74 | **Quantos cigarros você fuma por dia?** | **_____________**cigarros | **Q74** |
| 75 | **Você consome bebida(s) alcoólica(s)?** | 0( ) Não (**SE** **NÃO,** passa para a questão 78)  1( ) Sim. Com que idade?_______anos | **Q75** |
| 76 | **NESTA QUESTÃO MARQUE COM X TODAS AS RESPOSTAS CORRETAS**  **SE SIM,**  **Que bebida(s) alcoólica(s) você consome semanalmente?** | 0( ) Não consumo (**SE** **NÃO,** passa para a questão 78)  1( ) Grogue  2( ) Cerveja  3( ) Vinho  4( ) Outra bebida. Qual? ___________________ | **Q76** |
| 77 | **Você chega a ficar bêbado(a)?** | 0( ) Não  1( ) Sim | **Q77** |
| 78 | **NESTA QUESTÃO MARQUE COM X TODAS AS RESPOSTAS CORRETAS**  **Você já usou alguma dessas substâncias?** | 1( ) Padjinha  2( ) Cocaína  3( ) Craque  4( ) Êxtase  5( ) Outras. Qual? ______________  6( ) Nunca usei | **Q78** |

SÓ PARA QUEM TEVE RELAÇÕES SEXUAIS (RAPAZES E MENINAS) (perguntas de 79 a 105)

| 79 | **Hoje, você usa algum método anticoncepcional?** | 0( ) Não, por quê? ___________________  1( ) Sim, qual? ___________________ | **Q79** |
| --- | --- | --- | --- |
| 80 | **NESTA QUESTÃO MARQUE COM X TODAS AS RESPOSTAS CORRETAS.**  **Você tem intenção de usar um método de evitar a gravidez ou evitar doenças sexualmente transmissíveis ou SIDA na próxima relação sexual?** | 0( ) Não (**SE** **NÃO,** passa para a questão 98)  1( ) Sim  2( ) Ainda não pensei nisso | **Q80** |
| 81 | **NESTA QUESTÃO MARQUE COM X TODAS AS RESPOSTAS CORRETAS.**  **SE SIM,**  **Qual o meio de evitar a gravidez ou doenças sexualmente transmissíveis e SIDA que você pretende usar?** | 1( ) Pílula  2( ) Camisinh**a**  3( ) Injeção  4( ) Coito interrompido (gozar fora)  5( ) Tabelinha ou calendário  6( ) Outro, qual?_________  7( ) Não tem  8( ) Abstinência (não ter relações sexuais)  9( ) DIU  99( ) Não sei/não me lembro | **Q81** |
| 82 | **NESTA QUESTÃO MARQUE COM X TODAS AS RESPOSTAS CORRETAS.**  **Onde que você acha que alguém que estuda nesta escola pode conseguir o método que deseja para evitar a gravidez ou doenças sexualmente Transmissíveis e SIDA?** | 1( ) Com pai/mãe  2( ) Na farmácia/clínica particular  3( ) Em um posto de saúde público/hospital  4( ) Com amigos(as)  5( ) Em outro lugar. Onde?____________________  9( ) Não sei/não me lembro | **Q82** |
| 83 | **Nos últimos 6 meses, quantas vezes você teve relação Sexual?** | **___________________** | **Q83** |
| 84 | **Nos últimos 6 meses, com quantas pessoas você teve relação sexual?** | **_________________** | **Q84** |
| 85 | **Quando foi a sua última relação sexual?** | _____anos; ______meses  9( ) Não sei/não me lembro | **Q85** |
| 86 | **Com quem você teve a sua última relação sexual?** | 1( ) Namorado(a)  2( ) Amigo(a)  3( ) Parente  4( ) Noivo(a)  5( ) Marido/esposa  6( ) Outro. Quem? ___________________ | **Q86** |
| 87 | **Foi de comum acordo entre os dois?** | 1( ) Sim  2( ) Não  3( ) Eu forcei a situação  4( ) Ele/ela forçou a situação  5( ) Outro motivo, qual? ____________________________ | **Q87** |
| 88 | **NESTA QUESTÃO MARQUE COM X TODAS AS RESPOSTAS CORRETAS.**  **Qual foi a emoção mais importante que você sentiu na sua última relação sexual?** | 1( ) Dor  2( ) Medo  3( ) Felicidade/amor  4( ) Prazer  5( ) Outra. Qual? ___________________  9( ) Não sei | **Q88** |
| 89 | **Você acha que esta última relação sexual foi planejada ou simplesmente aconteceu**? | 1( ) Planejada  2( ) Aconteceu  3( ) Outro motivo, qual?_______________  9( ) Não sei | **Q89** |
| 90 | **Você conhecia métodos ou maneiras de evitar filhos ou de evitar uma doença sexualmente transmissíveis e SIDA na sua última relação sexual?** | 0( ) Não (**SE** **NÃO,** passa para a questão 98)  1( ) Sim | **Q90** |
| 91 | **NESTA QUESTÃO MARQUE COM X TODAS AS RESPOSTAS CORRETAS.**  **SE SIM,**  **Quais os métodos ou maneiras de evitar flhos ou de evitar uma doença sexualmente transmissível e SIDA que você já ouvia falar na sua última relação sexual?** | 01( ) Pílula  02( ) Camisinha  03( ) Injeção  04( ) Coito interrompido (gozar fora)  05( ) Tabelinha ou calendário  06( ) Outro. Qual? ___________________  07( ) Não ouviu falar  08( ) Abstinência (não ter relações sexuais)  09( ) DIU  99( ) Não sei/não me lembro | **Q91** |
| 92 | **Você usou algum método para evitar filhos ou evitar uma doenças sexualmente transmissíveis e SIDA na sua última relação sexual?** | 0( ) Não (**SE** **NÃO,** passa para a questão 97)  1( ) Sim | **Q92** |
| 93 | **NESTA QUESTÃO MARQUE COM X TODAS AS RESPOSTAS CORRETAS.**  **SE SIM,**  **Quais os métodos você utilizou na sua última relação sexual?** | 1( ) Pílula  2( ) Camisinha  3( ) Injeção  4( ) Coito interrompido (gozar fora)  5( ) Tabelinha ou calendário  6( ) Outro. Qual? ___________________  7( ) Não usou  8( ) Abstinência (não ter relações sexuais)  9( ) DIU  99( ) Não sei/não me lembro | **Q93** |
| 94 | **Quem tomou a iniciativa de usar este(s) método(s)?** | 1( ) Eu  2( ) Companheiro(a)  3( ) Nós dois  9( ) Não sei/não me lembro | **Q94** |
| 95 | **Você usou camisinha na sua última relação sexual?** | 0( ) Não (**SE** **NÃO,** passa para a questão 97)  1( ) Sim | **Q95** |
| 96 | **SE SIM,**  **Por que usou**? | 1( ) Para evitar a gravidez 2( ) Pode prevenir duplo propósito: gravidez e doenças sexualmente transmissíveis, incluindo SIDA3( ) Para evitar doenças sexualmente transmissíveis, incluindo SIDA 4( ) Não conhecia bem companheiro(a)  5( ) Outros motivos. Quais? ___________________  9( ) Não sei/não me lembro | **Q96** |
| 97 | **SE NÃO,**  **Por que não usou?** | 1( ) Não esperava ter relação sexual2( ) Queria engravidar3( ) Tinha vergonha4( ) Conhecia bem o(a) companheiro(a)5( ) Não gosta de usar6( ) Outros motivos. Quais? ____________________________ 9( ) Não sei/não me lembro | **Q97** |
| 98 | **NESTA QUESTÃO MARQUE COM V TODAS AS AFIRMAÇOES CORRETAS E MARQUE COM F TODAS AS AFIRMAÇÕES FALSAS.** | Q**uestões sobre conhecimento de métodos anticoncepcionais:** **1( ) Diafragma é descartável** **2  O diafragma é colocado dentro do útero****3  O diafragma deve ser retirado entre 8 e 12 horas após a relação sexual****4  A injeção pode alterar a menstruação****5  A pílula do dia seguinte deve ser tomada até 72h****6  O diafragma só deve ser usado com creme espermicida****7  A pílula diminui o sangramento menstrual****8  A injeção deve ser aplicada semanalmente****9  Quando termina uma caixa de pílula, deve-se começar outra no dia seguinte****10  O coito interrompido provoca dor de cabeça no homem****11  O coito interrompido é seguro para evitar filhos****12  A tabelinha é muito eficaz para evitar filho****13  Só quem tem ciclos regulares pode usar tabelinha****14  O DIU é colocado dentro do útero****15  Quando a mulher esquece de tomar pílula, não deve tomar mais durante o resto do mês** **16  A mulher deve tomar pílula todos os dias, sempre no mesmo horário****17  O DIU atrapalha a relação sexual****18  A camisinha masculina deve ser retirada quando o pênis ainda está ereto****19  A camisinha masculina serve só para evitar filhos** | **Q98** |
| 99 | **Em média, o intervalo entre o início de uma menstruação e o de outra é de:** | 1( ) 10 a 15 dias  2( ) 20 a 23 dias  3( ) 28 a 30 dias  4( ) 50 a 60 dias  9( ) Não sei | **Q99** |
| 100 | **Para uma mulher com ciclo menstrual normal, qual a época mais provável para engravidar?** | 1( ) Em torno de 7 dias antes do primeiro dia da menstruação  2( ) Em torno de 14 dias depois do início da menstruação  3( ) Durante a menstruação  4( ) Não sei | **Q100** |
| 101 | **Você já teve alguma doença transmitida pelo sexo (DST)?** | 0( ) Não  1( ) Sim  9( ) Não sei/não me lembro | **Q101** |
| 102 | **Você já pensou alguma vez em utilizar camisinha além de estar a usar outro método?** | 0( ) Não (**SE** **NÃO,** passa para a questão 104)  1( ) Sim | **Q101** |
| 103 | **NESTA QUESTÃO MARQUE COM X TODAS AS RESPOSTAS CORRETAS**  **SE SIM,**  **Qual é o motivo?** | 0( ) Não queria engravidar  1( ) Para evitar a gravidez **2( ) Em caso de rompimento da camisinha, para evitar a gravidez** 3( ) Em caso de rompimento da camisinha, além de evitar a gravidez evitar a transmissão de doenças sexualmente transmissíveis, incluindo vírus de SIDA ao bebe  4( ) Outros motivos  Quais? ___________________  9( ) Não sei/não me lembro | **Q103** |
| 104 | **NESTA QUESTÃO MARQUE COM X TODAS AS RESPOSTAS CORRETAS**  **Quando você acha que uma pessoa pode (deve) ter relações sexuais com outra?** | 1( ) Basta que tenham atração  2( ) Quando são namorados  3( ) Sejam pelo menos bons amigos  4( ) Apenas quando estão noivos  5( ) Só depois do casamento  9( ) Não sei | **Q104** |
| 105 | **NESTA QUESTÃO MARQUE COM X TODAS AS RESPOSTAS CORRETAS**  **Com quem você se sente à vontade para conversar sobre a sua vida sexual?** | 1( ) Meu pai  2( ) Minha mãe  3( ) Outro(s) parente(s)  4( ) Amigos(as)  5( ) Namorado(a)  6( ) Enfermeiro(a) do posto de saúde  7( ) Médico(a) do posto de saúde  8( ) Professor(a)  9( ) Outro. Quem? ___________________ | **Q105** |

SÓ PARA MENINAs COM INÍCIO DA VIDA SEXUAL

| 106 | **Você já ficou grávida?** | 0( ) Não (**FIM DO QUESTIONÁRIO**)  1( ) Sim | **Q106** |
| --- | --- | --- | --- |
| 107 | **SE SIM,**  **Qual foi a sua idade na primeira gravidez?** | ______ anos | **Q107** |
| 108 | **Nesta gravidez você fez/faz pré-natal?** | 1( ) Sim, número de consultas: ____________  2( ) Não, por quê? ____________________________  9( ) Não sei | **Q108** |
| 109 | **Qual foi sua idade no nascimento do seu primeiro filho?** | ______ anos | **Q109** |
| 110 | **Quantas vezes você ficou grávida?** | 1( ) Uma vez  2( ) Duas vezes  3( ) Mais de duas | **Q110** |
| 111 | **Você fez pré-natal na última gravidez?** | 0( ) Não, por quê? ___________________________  1( ) Sim, número de consultas _________ | **Q111** |
| 112 | **Todas as vezes em que você engravidou o bebê nasceu vivo?** | 0( ) Não, em qual não nasceu vivo?  1**^a^** gravidez  2**^a^** gravidez  3**^a^** gravidez    1( ) Sim | **Q112** |
| 113 | **Quanto(s) continua(m) vivo(s)?** | 1( ) Todos  2( ) Apenas o filho da primeira gravidez  3( ) Outra situação. Qual? ___________________ | **Q113** |
| 114 | **Você teve bebê que nasceu vivo nos últimos 12 meses?** | 0( ) Não  1( ) Sim | **Q114** |
